# Implementing the WHO rehabilitation competency framework in undergraduate medical education: a context-specific adaptation for neurorehabilitation training

**DOI:** 10.3389/fneur.2025.1704836

**Published:** 2025-12-15

**Authors:** Siyu Zhou, Yuanmingfei Zhang, Yanyan Yang, Jingyu Liu, Wenjing Qi

**Affiliations:** Department of Rehabilitation Medicine, Peking University Third Hospital, Beijing, China

**Keywords:** WHO, rehabilitation competency framework, rehabilitation education, undergraduate medical education, clinical training

## Abstract

**Background:**

The World Health Organization (WHO) released the Rehabilitation Competency Framework (RCF) in 2020, encompassing five core domains—Practice, Professionalism, Learning and Development, Management and Leadership, and Research—along with cross-cutting values and beliefs that establish international standards for rehabilitation education. However, current neurorehabilitation training in Chinese clinical undergraduate programs lacks a competency-oriented structure. For example, Peking University’s existing curriculum includes only 8 lecture h and an optional 2-week practicum, with assessment predominantly based on written examinations rather than competency evaluation.

**Methods:**

Curriculum Design: We employed a Delphi method (involving 3 rounds with 13 experts) to construct an RCF-based curriculum system, defining entry-level proficiency competencies (e.g., Practice P1-P4 and Core Values V1-V4). Implementation: We delivered an 8-h RCF theory plus 2-h case-based learning (CBL) to 36 eight-year program undergraduates. We conducted paired t-tests to evaluate competency changes before and after the intervention. The Rehabilitation Physician Competence Questionnaire was used as the core assessment tool. Paired t-tests were conducted to compare the 13 competence indicators before and after the curriculum reform to verify the effectiveness of the competence-oriented teaching method.

**Results:**

Curriculum Framework: Four modules—Professionalism, Foundations, Core Competencies, and Intensive Practicum—covering all RCF domains were developed. The Core Competencies module emphasized “Assessment Skills (P3)” and “Evidence-Based Decision Making (R1).” Outcomes: A total of 13 of 15 competencies showed significant improvement post-intervention (*p* < 0.05), including technical skills (+1.25 points) and empathy (+0.42 points). Research competency and patient advocacy showed non-significant changes.

**Conclusion:**

This study established a closed-loop system of “contextualized competency framework → curriculum development → multidimensional evaluation,” demonstrating RCF’s effectiveness in enhancing undergraduate rehabilitation competencies. It provides a paradigm for rehabilitation education aligned with Chinese characteristics.

## Background

The World Health Organization (WHO), in collaboration with global rehabilitation experts and international professional associations, pioneered the development of the Rehabilitation Competency Framework (RCF) in 2020 (1).This landmark initiative aims to address global rehabilitation workforce challenges by establishing standardized competency benchmarks. The RCF framework, which is also currently available in Chinese (2) through the official channels of the WHO, is structured around the following:.

Core Values and Beliefs: They serve as the foundational ethos that extends across all competency domains.

### Five domains of competency

- Rehabilitation Practice: Clinical skills and service delivery
- Professionalism: Ethical conduct and accountability
- Learning and Development: Continuous professional growth
- Management and Leadership: Healthcare system engagement
- Research: Evidence-based practice advancement (3)

Competency has emerged as a critical evaluation tool for personnel selection, education and training, and certification. From a professional perspective, competency frameworks primarily establish minimum standards for occupational practice. In North America, competency-based approaches have been systematically integrated into professional education curricula (4). In other professional fields, such as nursing, the British Nursing Council incorporates core competencies such as communication and ethical decision-making into the certification standards for registered nurses. However, this approach has the drawback of fragmented assessment methods. The competency model can construct a systematic ability framework and standardize core competencies. This model faces challenges such as insufficient localized theories and evaluation tools as well as generalized clinical practice assessments. Current neurorehabilitation training for clinical undergraduates in China lacks competency-oriented instructional content and assessment systems. For example, Peking University’s eight-year medical program offers merely 8 h of theoretical lectures and 2 h of case-based learning (CBL), followed by an optional 2-week clinical practicum without specific competency requirements. The assessment relies entirely on closed-book examinations testing factual knowledge, neglecting competency evaluation.

The primary objective of this study is to set the training goals for the core rehabilitation courses for undergraduate students majoring in clinical medicine, based on the RCF’s division into different competency domains. It aims to analyze rehabilitation competencies across five specific areas corresponding to the core values and beliefs in particular neurorehabilitation scenarios and integrate them into the corresponding undergraduate clinical medicine curriculum. Implementing education and training with competencies as the goal helps students acquire the corresponding job competency levels at each stage, laying a foundation for cultivating competent rehabilitation physicians.

## Methods

Initially, through literature reviews, on-site and online surveys, and interviews, the undergraduate courses of neurorehabilitation in both domestic and international settings, as well as the reform of RCF teaching in different specialties, were systematically examined. Based on these analyses, the expected forms of the basic competencies (entry-level standards) for rehabilitation physicians, the expected course objectives, the expected analysis of competencies, the core course contents, and the main knowledge units were formulated.

Subsequently, a Delphi method was applied in the field of neurorehabilitation medicine education for the above three items. Four medical education experts were selected, all of whom hold senior professorial or higher titles and have participated in national-level medical education reform projects. Two experts in RCF translation and application were also chosen, both of whom had participated in the localizing project of the WHO RCF framework and have published numerous papers related to competency models. Seven frontline educators in neurology and musculoskeletal rehabilitation were selected, all of whom hold the positions of director or deputy director of the rehabilitation departments in tertiary hospitals and have 10 years of clinical teaching experience. A total of 13 experts were invited for consultation. The first round of the questionnaire included open-ended questions, including the abovementioned three items, and instructions for filling them out. The experts’ opinions were anonymously summarized. A total of three rounds of questionnaires were sent out. The coefficient of variation (CV) in the third round was 0.21, and the recovery rates in all three rounds were ≥ 80%. The entire Delphi process was anonymized, and the interval between each round of questionnaire survey was 2 weeks.

**Figure 1:**
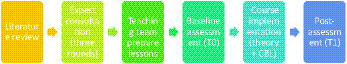
Methodology flowchart.

Finally, the teaching team conducted two rounds of focused lesson preparation on the core content of the teaching reform, revised the teaching outline centrally, and created new lecture slides and lesson plans. A total of 36 students from the 2021 cohort of the eight-year undergraduate program at Peking University were selected to conduct the baseline assessment using the Rehabilitation Physician RCF questionnaire (5). The Chinese version of this questionnaire has already been verified for its reliability and validity through the team’s previous research. Each ability is rated using the Likert 5-point scale (1 = completely does not conform, 5 = completely conforms). The same questionnaire was used for both the initial and final assessments, and a paired t-test was conducted based on the differences in the scores of the 13 ability indicators. Regarding the sample size estimation, an effect size of 0.5, an alpha level of 0.05, and a statistical power of 0.8 were set. The minimum sample size was calculated to be 34 cases, but 36 cases were actually included. Subsequently, the Shapiro–Wilk test was used, and all the ability indicators T0/T1 data satisfied the normal distribution assumption with a *p*-value of > 0.05. Subsequently, an 8-h RCF theory course and a 2-h CBL course were held. After the course, the rehabilitation physician competency questionnaire was administered again. Data analysis was performed using SPSS 23.0 software, with paired t-tests employed, and the mean differences along with 95% confidence intervals were reported. This study was approved by the Scientific Research Ethics Committee of Peking University Third Hospital (No. (2023) Med Ethics Review No. [542–01]). The methodology flowchart is shown in Figure 1.

## Results

Based on the theoretical framework of the International Classification of Functioning, Disability, and Health (ICF) and the perspective of functional rehabilitation, as well as guidance of the RCF, as well as relevant international educational certification standards, the basic competency characteristics for entry-level proficiency (minimum requirements) were determined through a Delphi expert process and are presented in Table 1.

**Table 1:** Expected forms of basic competencies (entry-level requirements) for rehabilitation physicians.

P (Practice)	PM (Professionalism)	Learning and Development (LD)	Management and Leadership (ML)	Research (R)	Core Values (V)	Belief (B)
P1 often works under the guidance of excellent rehabilitation workers P2 conducts treatment following a specific scenario rehabilitation treatment plan P3 has basic knowledge and skills for specific scenarios (at the undergraduate level) P4 can communicate with individuals with functional rehabilitation needs and their families	PM1 often works under the guidance of excellent rehabilitation workers PM2 formulates standard rehabilitation treatment work processes PM3 writes relevant records and reports for specific scenario rehabilitation treatment PM4 has basic knowledge and skills for specific scenario rehabilitation treatment	LD1 can provide various supports for team members LD2 can formulate personal professional development plans LD3 can regularly participate in specific scenario rehabilitation treatment, professional education, and training LD4 has the ability for lifelong learning and self-directed learning LD5 supports personal and peer professional development and learning	ML1 can contribute to the normal operation and development of the team ML2 can conduct scientific procurement and reasonable allocation of specific scenario rehabilitation treatment resources within the team ML3 can organize team members at the undergraduate level or below to carry out mass fitness, health education, and community rehabilitation guidance programs in the community	R1 applies specific scenario rehabilitation treatment practice guidelines to clinical decision-making and practice R2 evaluates practices using personal, team, and peer experiences and supports evidence-based conclusions R3 can participate in basic and clinical scientific research related to specific scenario rehabilitation treatment practice	V1 Compassion and empathy V2 Sensitivity and respect for differences V3 Human rights and dignity V4 Right to self-determination	B1 Good functional state B2 Personal/family-centered B3 Collaboration B4 Full coverage of needs

In the undergraduate teaching reform, it is emphasized that the educational goal of undergraduate professional education is to cultivate high-quality professionals who can initially provide comprehensive technical services. They should possess basic, reliable, professional, and effective knowledge of neurorehabilitation treatment and skills across multiple professional directions, as well as a certain medical humanistic spirit and dedicated professional attitude. Through the Delphi method, the expected correspondence between the disciplines, courses, and teaching objectives required for cultivating qualified rehabilitation physicians (undergraduate education) is shown in Table 2.

**Table 2:** Analysis of expected course objectives and competency expectations for undergraduate rehabilitation medicine programs based on RCF.

Course content classification	Course system classification	Assessment ratio	Teaching objectives	Capability characteristics
Quality Education	Theory Courses (8 class h in total, the same below)	10%	Possess basic theoretical knowledge in humanities, social sciences, and natural sciences; Have a correct worldview, outlook on life, and set of values and possess a high sense of social responsibility; Be able to communicate in a foreign language and read basic professional foreign-language materials; Have basic information literacy; Have the basic essentials and skills for physical exercise and be physically strong; Have basic safety concepts and capabilities; Have good mental health, a sound personality, abide by laws and regulations, and engage in innovation based on evidence; Have design, creation, and aesthetic abilities; Have correct self-awareness and good interpersonal relationships, respect colleagues and peers, and be able to work in teams.	Practice: P4; Professional Spirit: PM3; Learning and Development: LD1, LD2, LD4, LD5; Management and Leadership: ML1 Research: R2; Core Values: V1, V2, V3, V4; Beliefs: B1, B3
Professional Foundation Section	Theory Courses	20%	Have basic theoretical knowledge in clinical medicine and rehabilitation medicine; possess the professional theories and knowledge that rehabilitation physicians should have, such as those related to biology, behavior, society, psychology and clinical science in rehabilitation therapy; be able to conduct diagnoses of functional impairments, pathological analyses, imaging analyses, analysis of movement disorders, analysis of functional limitations, etc.; have knowledge related to policies and laws in rehabilitation therapy; be able to attempt to carry out evidence-based research by combining the cutting-edge clinical rehabilitation therapy.	Practice: P4; Professionalism: PM3, PM4; Learning and Development: LD1, LD2, LD4, LD5; Management and Leadership: ML1, ML3 Research: R2, R3; Core Values: V1, V2, V3, V4; Beliefs: B1, B2, B3
The core part of the profession	Theory Courses and CBL classes (totaling 2 class hours)	70%	Be able to assess health problems caused by factors such as cognitive impairment, physical functional impairment, speech and language, swallowing disorders, daily living ability impairment, and social participation impairment under guidance and be able to do so through standardized assessment; possess basic clinical knowledge of common and frequently-occurring diseases and initially master the principles and various methods of comprehensive rehabilitation treatment; be able to formulate high-quality rehabilitation treatment prescriptions with evidence-based medical support through reading, learning, and guidance.	Practice: P1, P3, P4; Professionalism: PM1, PM2, PM3, PM4; Learning and Development: LD1, LD2, LD3 Management and Leadership: ML1, ML3 Research: R1, R2, R3; Core Values: V1, V2, V3, V4; Belief: B2, B3
Professional practice	Rehabilitation internship (about 3 weeks)	0	By adhering to the patient-centered treatment principles, under the premise of establishing effective communication, respecting the impacts on the rehabilitation of service recipients caused by individual differences, cultural beliefs, and customs, advocating for the formulation of treatment plans based on the patients’ interests, the patients’ own or their family members’ wishes, implementing effective rehabilitation treatments under guidance, etc.—these measures can help patients reintegrate into society.	Practice: P1, P2, P3, P4; Professionalism: PM1, PM2, PM3, PM4; Learning and Development: LD1, LD3, LD5; Management and Leadership: ML1, ML2, ML3 Research: R1, R2, R3; Core Values: V1, V2, V3, V4; Belief: B2, B3, B4

The core course content includes basic knowledge of the nervous and muscular systems, as well as common internal and surgical diseases in rehabilitation. The fundamental theories that underpin rehabilitation therapy should be applied in practice and integrated with skill development and practical educational experiences. Clinical science is applied throughout the course content to support evidence-based practice and research in rehabilitation: evidence-based practice, types of data, literature search and review, research methods (qualitative, quantitative, mixed methods), application of statistics, literature evaluation, and research evaluation. The primary knowledge units include rehabilitation treatment concepts, ICF, rehabilitation assessment, assessment and intervention of physical health, physical factor therapy, rehabilitation of neurological diseases, rehabilitation of neuromusculoskeletal diseases controlled by the nervous system, rehabilitation of spinal cord injuries, community and health education, human motor developmentology, functions of Physical therapist (PT), occupational therapist (OT), speech therapist (ST) and orthotist (PO) in health and work, physical therapy, occupational therapy, therapeutic environment and assistive technology, speech-language and swallowing disorder treatment, and orthotic device fabrication, among others. See Table 3.

**Table 3:** Core curriculum contents and main knowledge units of rehabilitation medicine undergraduate program based on RCF.

Type	Teaching content	Core course content	Main knowledge units
Theory	Rehabilitation Therapy Techniques	Musculoskeletal and neuromuscular systems, as well as common internal and surgical diseases in rehabilitation	Exercise therapy, occupational therapy, therapeutic environment and assistive technology, speech-language and swallowing disorder treatment, and orthotic fabrication
	General Rehabilitation Medicine and Rehabilitation Assessment		Rehabilitation treatment concepts, ICF, rehabilitation assessment, community and health education, human motor developmentology, and PT&OT&ST&PO in health and work
	Orthopedic Rehabilitation		Rehabilitation for skeletal joint and muscle diseases and spinal cord injury rehabilitation
	Nerve Rehabilitation		Rehabilitation for neurological diseases
CBL	Rehabilitation during the perioperative period of artificial knee joint replacement	The basic theories supporting rehabilitation treatment should be applied in practice and combined with skill development and practical educational experience. Critical thinking, clinical reasoning, ethical conduct, professional skills, good communication, cultural responsiveness, good teamwork, client-centered care models, client assessment including outcome assessment, interpretation of assessment results and intervention plans, evidence-based intervention measures (such as physical therapy and physical activities, manual therapy, physical factor therapy, speech-language therapy, occupational therapy, etc.), digital technology practice, health promotion and disability prevention, education, and so on.	Postoperative rehabilitation assessment for Total Knee Arthroplasty (TKA), prevention of complications, formulation of rehabilitation treatment plans, PT & OT therapeutic techniques
Internship	Selected Course Internship for Eight-Year Program	Clinical science is applied throughout the entire life cycle to support evidence-based practice and research in rehabilitation therapy: evidence-based practice, types of data, literature search and review, research methods (qualitative, quantitative, and mixed methods), application of statistics, literature evaluation, and research evaluation.	Practical educational experience: Rehabilitation physicians provide comprehensive client management in a variety of outpatient and inpatient settings (including assessment, examination, diagnosis, and prognosis determination), care plans, intervention contents (including treatment, education, prevention, health promotion and health planning, leadership, management, and evaluation)

This study adopted a pre-post control design to evaluate the impact of the teaching reform intervention for RCF undergraduate students on the competence of rehabilitation physicians. Baseline data (T0) and post-intervention data (T1) were analyzed using paired t-tests. All 36 students underwent two assessments, with a data recovery rate of 100%. After the intervention, all 15 indicators except for respecting and protecting the patient’s interests, scientific research awareness, and ability showed significant improvement. See Table 4 for details.

**Table 4:** Paired *t*-test of the competency scale for rehabilitation physicians.

Indicator	Baseline (T0)	Post-intervention (T1)	MD (95% CI)	*p*-value
Compassion and empathy	3.722 ± 0.779	4.139 ± 0.639	−0.417(−0.153—0.680)	0.009
Sensitivity and respect for diversity	4.028 ± 0.654	4.444 ± 0.504	−0.417(−0.195—0.638)	0.004
Dignity and human rights	4.222 ± 0.637	4.556 ± 0.504	−0.333(−0.118—0.549)	0.016
Self-determination	4.306 ± 0.525	4.444 ± 0.558	−0.139(0.039—0.316)	0.230
Basic theories and knowledge of rehabilitation assessment and their application	2.417 ± 0.937	3.667 ± 0.632	−1.250(−0.933—1.567)	<0.001
Rehabilitation technical level	2.333 ± 1.042	3.222 ± 0.929	−0.889(−0.536—1.241)	0.001
Observation of the condition and ability to narrate	2.944 ± 0.893	3.750 ± 0.692	−0.806(−0.504—1.108)	<0.001
Emergency handling and anticipation ability	2.611 ± 0.964	3.417 ± 0.841	−0.806(−0.479—1.132)	0.001
Service capability	3.500 ± 0.971	4.028 ± 0.696	−0.528(−0.199—0.856)	0.026
Respect and protection of patients’ interests	4.028 ± 0.736	4.333 ± 0.586	−0.306(−0.056—0.555)	0.062
Interpersonal relationships	3.528 ± 0.878	4.000 ± 0.586	−0.472(−0.175—0.769)	0.009
Application of knowledge ability	2.944 ± 1.040	3.722 ± 0.701	−0.778(−0.426—1.130)	<0.001
Critical thinking ability and flexibility	3.139 ± 1.018	3.806 ± 0.710	−0.667(−0.322—1.011)	0.002
Continuous learning ability	3.500 ± 0.941	3.972 ± 0.810	−0.472(−0.154—0.791)	0.045
Teamwork and leadership ability	2.889 ± 1.166	3.528 ± 0.810	−0.639(−0.244—1.033)	0.010
Resource management ability	3.056 ± 1.040	3.667 ± 0.756	−0.611(−0.259—0.963)	0.004
Research awareness and ability	3.028 ± 1.158	3.472 ± 0.878	−0.444(−0.052—0.836)	0.107

## Discussion

The RCF framework provides significant guidance for undergraduate neurorehabilitation education. This study is the first to integrate the five domains of RCF—Practice, Professional Spirit, Learning and Development, Management and Leadership, and Research—and core values/beliefs into the curriculum design of clinical medicine undergraduate courses in China. As shown in Table 1, the course content forms a clear mapping with the competency characteristics. For example, the core part of the course focuses on cultivating “rehabilitation assessment ability (P3)” and “evidence-based decision-making ability (R1),” which compensates for the deficiency of traditional closed-book examinations in ignoring ability evaluation. This study refers to the rehabilitation education certification systems of countries such as the United States (6) and Australia (7). Brandstater (8) conducted a survey on the training and certification of rehabilitation physician positions in 45 countries and regions across the world. The results showed that 43 countries provided training, and 41 countries offered certification examinations. However, the training systems and certifications varied. The Canadian Rehabilitation Therapy Advisory Committee (9) leads the certification of rehabilitation therapy education in Canada, covering basic and entry-level abilities required for a career, including 7 domains (professional knowledge, communication, collaboration, management, leadership, academic ability, and professional spirit), with approximately 140 requirements for abilities. This teaching reform sets the primary proficiency as the undergraduate training goal, which is consistent with the concept of stratified training of rehabilitation human resources proposed by the WHO. In particular, international consensus, such as “collaborative rehabilitation (B3)” and “comprehensive coverage of needs (B4),” is included in the belief cultivation, reflecting the global trend of rehabilitation services.

The data after the intervention revealed that, among the 15 indicators of competence, 13 showed significant improvement (*p* < 0.05). Among them, the technical ability indicators showed an increase of 1.25 points in rehabilitation skills (95% CI: 0.93–1.57), reflecting the effectiveness of practice-oriented teaching; the indicators of humanistic quality showed an increase of 0.42 points in compassion and empathy (*p* = 0.009), confirming the necessity of values education. However, although scientific research awareness and patient interests were identified as key areas to be strengthened during the lesson plan preparation for this teaching reform,improvements in scientific research awareness and ability (*p* = 0.107) and the protection of patient interests (*p* = 0.062) still did not reach a significant difference. This might be related to the insufficient duration of undergraduate scientific research practice and the relatively low proportion of clinical ethics case teaching. It is necessary to strengthen these aspects in the subsequent teaching reform.

This study has some limitations. The current research only included 36 eight-year program students. Due to ethical restrictions, no parallel control group was set up, and only a self-control comparison before and after the course was conducted. Moreover, there was a lack of post-graduation competency tracking data. Subsequent multi-center large-sample studies can be carried out, and an alumni database can be established for long-term effect evaluation. A multi-disciplinary collaboration is emphasized by the RCF, but the existing courses are still dominated by teachers from the rehabilitation medicine department. In the future, collaboration with psychology, social work, and other disciplines can be jointly developed to create interdisciplinary modules, further strengthening the core concept of “patient-centeredness (B2).” Based on the team’s previous experience in Massive Open Online Courst (MOOC) construction, it is recommended to develop an RCF competency digital profiling system. Through AI analysis of learning behaviors and their correlation with competency development, personalized training can be achieved (10).

## Conclusion

This study systematically reformed the neurorehabilitation courses for clinical undergraduate students based on the World Health Organization’s Rehabilitation Competence Framework (RCF). Through expert consensus using the Delphi method, the five major domains of RCF—Practice, Professional Spirit, Learning and Development, Management and Leadership, and Research— and core values/beliefs were integrated into the curriculum design of Chinese clinical medical undergraduate programs, forming a closed-loop system of “scenario-based competence framework → curriculum development → multi-dimensional evaluation,” with remarkable teaching effects.

## Data Availability

The raw data supporting the conclusions of this article will be made available by the authors, without undue reservation.
